# Evidence synthesis for constructing directed acyclic graphs (ESC-DAGs): a novel and systematic method for building directed acyclic graphs

**DOI:** 10.1093/ije/dyz150

**Published:** 2019-07-19

**Authors:** Karl D Ferguson, Mark McCann, Srinivasa Vittal Katikireddi, Hilary Thomson, Michael J Green, Daniel J Smith, James D Lewsey

**Affiliations:** 1 MRC / CSO Social and Public Health Sciences Unit, University of Glasgow, Glasgow, UK; 2 Mental Health and Wellbeing, University of Glasgow, Glasgow, UK; 3 Health Economics and Health Technology Assessment, University of Glasgow, Glasgow, UK

**Keywords:** Directed acyclic graphs, evidence synthesis, research methods, counterfactual causal inference

## Abstract

**Background:**

Directed acyclic graphs (DAGs) are popular tools for identifying appropriate adjustment strategies for epidemiological analysis. However, a lack of direction on how to build them is problematic. As a solution, we propose using a combination of evidence synthesis strategies and causal inference principles to integrate the DAG-building exercise within the review stages of research projects. We demonstrate this idea by introducing a novel protocol: ‘Evidence Synthesis for Constructing Directed Acyclic Graphs’ (ESC-DAGs)’.

**Methods:**

ESC-DAGs operates on empirical studies identified by a literature search, ideally a novel systematic review or review of systematic reviews. It involves three key stages: (i) the conclusions of each study are ‘mapped’ into a DAG; (ii) the causal structures in these DAGs are systematically assessed using several causal inference principles and are corrected accordingly; (iii) the resulting DAGs are then synthesised into one or more ‘integrated DAGs’. This demonstration article didactically applies ESC-DAGs to the literature on parental influences on offspring alcohol use during adolescence.

**Conclusions:**

ESC-DAGs is a practical, systematic and transparent approach for developing DAGs from background knowledge. These DAGs can then direct primary data analysis and DAG-based sensitivity analysis. ESC-DAGs has a modular design to allow researchers who are experienced DAG users to both use and improve upon the approach. It is also accessible to researchers with limited experience of DAGs or evidence synthesis.

**Key Messages**

- We present a novel method—Evidence Synthesis for Constructing Directed Acyclic Graphs (ESC-DAGs)—to answer the call for a systematic approach to building DAGs.
- ESC-DAGs is a theory-driven approach to building DAGs from the empirical literature with a strong emphasis on transparency.
- DAGs produced from ESC-DAGs are summative representations of the literature that can be used to direct data analysis, and for DAG-based sensitivity analysis.
- This demonstration article is accessible to researchers with limited to advanced experience of DAGs.
- ESC-DAGs has a modular design allowing experienced DAG users to both apply and improve upon the approach incrementally.

## Background

Causal inference methods are popular in observational research, with directed acyclic graphs (DAGs) being notably prominent.^1^ A DAG posits causal relationships between variables as arrows between nodes. Absence of an arrow between nodes indicates no causal effect, and nodes can be measured or unmeasured.^2–4^ Any variable that influences at least two others should be included. DAGs use the ‘backdoor criterion’, a mathematical ruleset, to determine which variables should be controlled for when estimating the effect of one on another. DAGs are thus valuable tools for guiding analysis^5^ (see Supplementary Appendix 1, available as Supplementary data at *IJE* online, for more detail on basic DAG concepts). However, applied DAG use has often been problematic. In a recent review of use, Tennant *et al*. note a ‘huge variation in practice’,^6^ finding that: DAGs are often overly simplistic; are altered to fit available data; and are regularly not presented in studies using them.^7^ They conclude that there is a lack of ‘guidelines for best practice’ for DAG construction and use, echoing earlier calls for ‘a disciplined approach to developing DAGs’.^8^

While no systematic guidelines for DAG construction currently exist, there is loose consensus on at least three points. First, that theory and ‘background knowledge’ should have a central role in DAG construction.^8^^,^^9^ Key authors in causal inference have strongly argued the importance of ‘background knowledge’ for constructing DAGs.^10–13^ Second, no connection between two nodes in a DAG (claiming no relationship) is a stronger assertion than including one.^4^ This implies that researchers should ‘work backwards’ from a ‘saturated’ DAG—one in which all variables are inter-connected—and only delete connections that are thought impossible.^6^ Third, while data-driven methods such as stepwise selection are widely used and may offer value to building DAGs, they can induce bias by mistakenly adjusting for mediators or colliders.^14^

This article introduces ‘Evidence Synthesis for Constructing Directed Acyclic Graphs’ (ESC-DAGs), a novel review methodology proposing a principled approach to DAG construction. It marries the rigorous systemisation of evidence synthesis protocols (e.g. Cochrane systematic reviews^15–19^) with causal thinking as expressed in the potential outcomes framework (POF).^2^ ESC-DAGs systemises how background knowledge is used for determining which variables and connections between variables are included (working backwards from a saturated DAG). The approach leverages the empirical literature, first translating empirical findings into DAGs, and then synthesising these into one or more ‘integrated’ DAGs.

As a review method, ESC-DAGs is applied to studies identified by a literature search. Research projects using ESC-DAGs will already have completed a literature search corresponding to a well-defined research question (for example, according to PICO/PECO guidelines of Population, Intervention/Exposure(s), Comparison, Outcome^20^). Ideally, this would be a novel systematic review or a review of systematic reviews. However, because ESC-DAGs can produce highly complex DAGs with dozens of variables, systematic searches should be limited to the research question’s focal relationship(s) (e.g. exposure–outcome etc.). ESC-DAGs is designed to be useful to researchers with limited to advanced experience of DAGs. This article demonstrates ESC-DAGs didactically with examples of hypothetical and real studies from literature on adolescent alcohol use.

## ESC-DAGs methodology

Table 1 summarises the protocol for ESC-DAGs. Each study identified from a literature search goes through core processes of ‘mapping’, ‘translation’ and ‘integration’. The mapping process produces a DAG representing the conclusion of the study. Translation involves assessing the causal characteristics of each connection (referred to as ‘directed edges’ in the literature) of this DAG. Directed edges are compiled into an index (Supplementary Appendix 2, available as Supplementary data at *IJE* online). The integration stage combines directed edges from the index into one diagram. The final output of the process is one or more ‘integrated DAGs’ (I-DAGs). Decision-making is recorded in a ‘decision log’, to be provided as an appendix to ESC-DAGs (Supplementary Appendix 3, available as Supplementary data at *IJE* online). In this article: the term ‘study’ refers to published articles included in the ESC-DAGs process; ‘researcher(s)’ to the authors of those studies; and ‘reviewer(s)’ to ESC-DAGs users.

**Table 1.** Summary of ESC-DAGs protocol

Stage	Purpose	Process
Mapping	To apply graph theory to the conclusions of each study. This creates an ‘implied graph’ (IG) which acts as a transparent structural template for translation into a DAG.	Outcome variable of interest is set as DAG outcome(s). Exposure variable(s) of interest is set as DAG exposure(s). A directed edge is drawn originating from the exposure(s), terminating at the outcome(s). All control variables are entered as unassigned variables. A directed edge is drawn originating from each control to the exposure(s) and outcome(s). Mediators, instrumental variables etc. are mapped as per the study’s conclusions. The IG is saturated by drawing directed or undirected edges between all confounders (direction does not matter until the translation stage). The recombination process can be performed at this stage to help simplify an overly complex IG.
Translation	To apply causal theory to each relationship in the IG. This creates the DAG for the study. Each relationship in the IG is assessed under sequential causal criteria and a counterfactual thought experiment (See causal criteria sections in the text for detailed discussion).	The posited relationship and its reverse are both assessed. Edges may be retained as posited, reversed, or as bi-directional. If not, they are deleted. All retained edges are entered into the directed edge index. Temporality—does the posited cause precede effect? (If ‘yes’, proceed to next criterion. If not, assess reverse relationship.) Face-validity—is the posited relationship plausible? (If ‘yes’, proceed to next criterion. If not, assess reverse relationship.) Recourse to theory—is the posited relationship supported by theory? (Always proceed to the counterfactual thought experiment.) Counterfactual thought experiment—is the posited relationship supported by a systematic thought experiment informed by the POF? (Once completed, always assess the reverse relationship unless already assessed.)
Integration 1: synthesis	To combine the translated DAGs into one by synthesising all indexed directed edges.	A new DAG is created to serve as the integrated DAG (I-DAG). The focal relationship is added to the I-DAG (as per mapping steps 1–3). Each indexed directed edge pertaining to the focal relationship (including its corresponding node) is added to the diagram. Each indexed directed edge pertaining to other nodes is added (e.g. between confounders). Conceptually similar nodes should be grouped together in virtual space to aid the recombination process.
Integration 2: recombination	To combine nodes for either practical reasons (i.e. to reduce complexity) or substantive reasons (i.e. to establish consistency).	Is there theoretical support for combining two variables/nodes? Do the conceptually related nodes have similar inputs and outputs (i.e. do they ‘send to’ and ‘receive from’ the same nodes)?

### Mapping

The mapping process is intended to produce a DAG that corresponds to the conclusions of the study under review (accurate or otherwise) and is ‘saturated’ with an edge between all pairs of nodes. We refer to the output as that study’s ‘implied graph’ (IG). Table 1 describes how the mapping procedure converts study conclusions into DAGs.

#### Worked example

**Figure 1.**
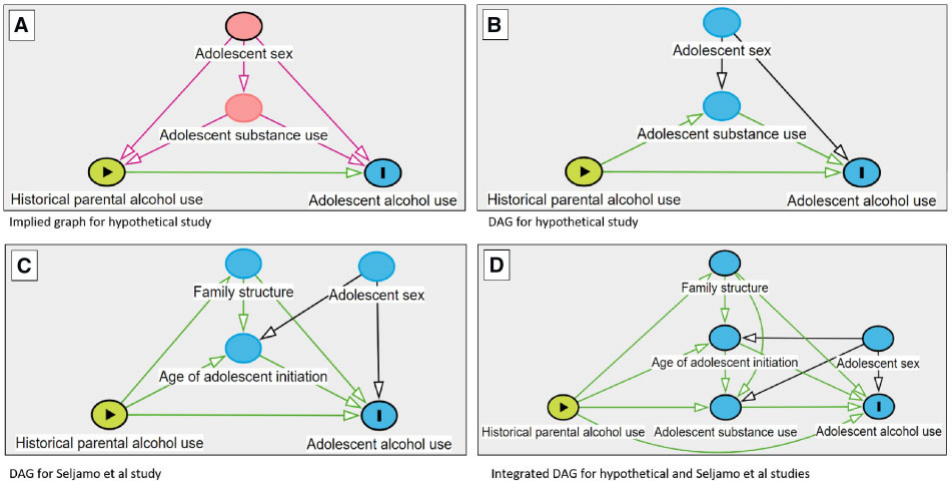
ESC-DAGs translation processes.

Take a hypothetical study where researchers were interested in effects of historical parental alcohol use on their offspring’s adolescent alcohol use and controlled for adolescent sex and substance use. Based on regression coefficients and confidence intervals, the researchers concluded that historical parental alcohol use was associated with adolescent alcohol use. The mapping process begins by entering the exposure (historical parental alcohol use) and outcome (adolescent alcohol use) into the diagram and then draws a directed edge from exposure to outcome. Next, the study’s control variables are added to the IG and directed edges are drawn from both to the exposure and outcome, assigning them as mutual causes of the exposure and outcome—confounders. This is because the POF definition of what should be controlled is a node that opens a backdoor path from the outcome to the exposure,^2^^,^^3^ and a confounder is the simplest way to represent this in an IG. The mapping process is intended to build a saturated DAG, so edges should also be drawn between all confounders, although directionality of inter-confounder edges is not important at this stage. The mapping stage is thus completed and the IG for the study is shown in Figure 1(A). Note that the recombination processes described below can also be applied at this stage if IGs become overly complex.

### Translation

With the IG formulated, reviewers next assess whether each posited connection is feasible, thus ‘translating’ the IG into a DAG. Numerous approaches are possible. Here, we propose and demonstrate a sequential causal criteria approach culminating in a counterfactual thought experiment. We recommend that alternative approaches place similar emphasis on explicating reviewers’ causal thinking in a structured and transparent way. Extensive discussion on the utility of causal criteria is available elsewhere.^21–23^

Each directed edge in the IG is assessed for three causal criteria: temporality; face-validity; and recourse to theory. They are primarily informed by the classic Bradford Hill viewpoints,^24^ and are compatible with the ‘inference to the best explanation’ approach advocated by Krieger and Davey Smith.^1^ If a relationship is determined to possess each criterion, a counterfactual thought experiment derived from the POF is used to further explicate the reviewers’ assumptions.^25^ The translation process thus combines ‘classic’ and ‘modern’ causal thinking and understands DAGs as ‘conceptual tools’^1^ for exploring causation, rather than substitutes for careful causal thinking.

The ESC-DAGs causal criteria operate sequentially, with each criterion designed to elaborate over the previous. If any criterion on the edge is not present, the edge can be deleted. The exception is the recourse to theory criterion—absence of theory in the study or according to the reviewer does not equate to absence of effect.^26^ The counterfactual thought experiment is performed after assessing all criteria. All retained directed edges are entered into the directed edge index. However, each edge should be tested in both directions (i.e. with the head and tail of the arrow swapped). If the posited and reverse edges are both retained, then the relationship should be noted as bi-directional in the directed edge index. Reviewers can also note low confidence in particular directed edges.

#### Causal criterion 1—temporality

Of the Bradford Hill criteria, temporality is the only one not requiring extensive qualification or not yet disproven.^27^ It states that effect cannot precede cause. For example, in Figure 1(A), adolescent substance use cannot precede historical parental alcohol use, so the relationship would not be temporal. Unless the directed edge is not temporal, we proceed to causal criterion 2.

#### Causal criterion 2—face-validity

Face-validity is related to the Bradford Hill criterion of (biologic) plausibility. Nested within the wider causal criteria scheme, the face-validity criterion is a rapid means of using reviewer background knowledge to identify implausible relationships, given the temporality established in criterion 1. For example, in Figure 1(A) it is plausible that directed edges originate from sex, but implausible that historical parental alcohol use could influence adolescent sex assignment despite temporal ordering.

#### Causal criterion 3—recourse to theory

The recourse to theory criterion considers background and expert knowledge more overtly. It subsumes the temporality and face-validity criteria and continues to cement a platform for the counterfactual thought experiment. Where the face-validity criterion is concerned with the researcher’s own knowledge, the step assesses whether there is formal theoretical support for the relationship. The decision log for this criterion requires the reviewer to state briefly what theory applies (if any) with space for a reference. As noted above, lack of theory does not equate to lack of effect. As such the purpose of this criterion is not so much falsification as preparation for the next step.

#### Counterfactual thought experiment

Fundamentally, potential outcomes compare the outcome that would have occurred if all of the sample had been exposed, with the outcome that would have occurred if all of the sample had not been exposed.^3^^,^^4^^,^^25^ The counterfactual thought experiment employs this heuristic in a formulaic and transparent way, comparing two or more ‘counterfactual exposures’ and considering whether their potential outcomes would be different, given the causal criteria. The original study’s measurement of variables should be emulated.

Take the example of the effect of adolescent sex on adolescent alcohol use from Figure 1(A). Sex is the exposure node in this example and is a binary variable with values 1 (male) and 0 (female). The counterfactual exposure would therefore be all participants ‘set’ to male (or female). The study measures the outcome of adolescent drinking as frequency of use. The counterfactual thought experiment then hinges on the question of whether we would expect equivalent average scores between the potential outcome for counterfactual exposure 1 (male) and the potential outcome for counterfactual exposure 0 (female). Based on the temporality, face-validity, recourse to theory, and causal thinking encouraged by the counterfactual thought experiment, we hypothesise that the frequency of alcohol consumption (the potential outcomes), would differ between the two counterfactual exposures (sex) and so draw a directed edge from sex to adolescent alcohol use. Discussion on using counterfactuals for non-modifiable risk factors is available elsewhere.^2^^,^^28^

The directed edge from adolescent sex to adolescent alcohol use would be retained and indexed. This is the most common result. Directed edges can also be reversed or deleted (see Supplementary Appendix 2, available as Supplementary data at *IJE* online, for an example of how to structure counterfactual thought experiments in a decision log). For each study, the final DAG is produced once each directed edge (and its reverse) from the IG has been assessed under the causal criteria and counterfactual thought experiment, and the IG has been altered accordingly. In the case of this hypothetical example, the DAG would be equivalent to Figure 1(B). All directed edges included in the DAG are indexed for use during the integration process.

This simple example illustrates how the translation process retains, reverses and deletes directed edges implied by the assessed studies. The IG in Figure 1(A) is typical of many studies in that it over-controls some covariates. Firstly, adolescent sex does not confound the relationship between historical parental alcohol use and adolescent alcohol use, rather it is a risk factor on another pathway (thus, adjusting for it may increase the precision of the estimate, rather than remove any bias). More consequential would be controlling for adolescent substance use as a mediator, as this controls for part of the effect we are trying to estimate.^29^ Note that it would induce collider bias between historical parental alcohol use and adolescent sex.^2–4^

### Integration 1—synthesis

Synthesis integrates each directed edge from the directed edge index rather than combining the DAGs themselves. Once a DAG has been produced for all studies, each directed edge and its corresponding nodes are added to a blank DAG until the directed edge index is exhausted. The output is the I-DAG for the research question. Several practicalities are worth noting. First, the order in which directed edges are synthesised is not important—the I-DAG will be equivalent. Second, with each new node that is introduced, some directed edges that are not yet in the index will become possible and these should be assessed using the same procedures as were used during the translation process. Last, once the directed edge index has been exhausted, any variables that were not identified in the literature search but are believed to be important by the reviewers should be added as new nodes to the I-DAG. The mapping and translation steps should then be applied to these variables in the context of the I-DAG, as opposed to a new IG.

Synthesis is illustrated here on the DAG from the hypothetical study above and an empirical study by Seljamo *et al*.^30^ They were interested in parental predictors of adolescent alcohol use. They used multiple regression models with early adolescent alcohol initiation, family structure, adolescent sex, and historical parental alcohol use as explanatory variables. The DAG produced from the corresponding IG is presented in Figure 1(C). Each directed edge was entered into the directed edge index. Figure 1(D) is the I-DAG for the hypothetical and Seljamo studies. Each directed edge from both studies was entered into a new diagram. This DAG was then saturated, and all ‘new’ relationships were put through the translation process (for example the relationship between family structure and adolescent sex was rejected whereas age of alcohol initiation was hypothesised to cause other substance use). This I-DAG determines that the exposure–outcome relationship of historical parental alcohol use on adolescent alcohol use is unconfounded by any of the suggested covariates. In practice I-DAGs are likely to be much more complex than this simple demonstrative example.

### Integration 2—recombination

As the I-DAG grows more complex through further synthesis, it may be efficient to consider ‘recombining’ multiple similar nodes. This is possible because different studies commonly conceptualise the same construct in similar ways. There are at least two indications that recombination may be acceptable for any two nodes. The first is theoretical support, e.g. if both nodes are categories of another concept (parental monitoring and autonomy granting as categories of parenting practice) or if they are used interchangeably in the literature. A second indication is if nodes have identical directed edge input and output (i.e. receive from/send to the same nodes). If they feature different directed edges, then there is less support for recombining them.

## Discussion

Perhaps the most obvious strength of ESC-DAGs is how the resulting I-DAGs closely align with the fundamental purpose of DAG-based analysis—to identify appropriate adjustment strategies.^3^^,^^4^ I-DAGs may be used to direct analysis in an immediate sense. For mediation analysis, for example, they identify which confounders can be adjusted for conventionally and which require more advanced techniques (e.g. exposure-induced mediator-outcome confounders^31–33^). I-DAGs can also direct sensitivity analysis by testing the robustness of results to changes in the presence or direction of specific edges in the I-DAG. Clear candidates include relationships in which reviewers have less confidence or that were marked as bi-directional in the directed edge index. Another key strength is how the translation process balances the need for rigorous scrutiny with time and resource efficiency.

ESC-DAGs has been designed to work as a discrete but modular application. The above translation strategy—sequential causal criteria culminating in a counterfactual thought experiment—is far from definitive. There are numerous viable alternatives that can be ‘swapped in’; of particular note are the ROBINS-I and upcoming ROBINS-E tools.^34^ Relatedly, as with any evidence synthesis method, the role of subjectivity demands careful thought. The approach laid out in the ESC-DAGs methodology is to make the completion and later presentation of a decision log a key part of the process—in other words to emphasise transparency in lieu of objectivity.

The approach is not without limitations. For example, no matter what method is used for building a DAG from observational studies, the true causal structures may be missed. Relatedly, it must be noted that I-DAGs rely on theory more than evidence for much of the confounding structure. Further, even with the modular design, conducting an ESC-DAGs review is far from a trivial task. Moreover, several valuable extensions to the method are possible but would add further to an already labour-intensive set of tasks.

One extension could be further development of ESC-DAGs as the basis for systematic sensitivity analysis. For example, integrating one of the many tools available for assessing risk of bias in observational studies could help systemise the level of confidence reviewers have in particular relationships.^35^ Further, an I-DAG can be refined with algorithmic and data-driven techniques. For example, DAGitty (web-based software for creating, editing and analysing causal models) can be set to estimate either total or direct effects, indicating directed edges that might be superfluous to estimating the exposure’s effect on the outcome, and the R-DAGitty package uses partial correlations to test the conditional and unconditional independencies in a DAG to evaluate how consistent a DAG is with the dataset it is intended to represent.^36^ Additionally, the emphasis on transparency aligns neatly with the growing open science movement. For example, the decision log and code for the I-DAGs (i.e. from DAGitty.net) could be uploaded to version-control platforms, such as ‘github’.^37^

## Conclusion

Lack of direction in how DAGs are built has been identified as cause for concern.^1^^,^^6^^,^^8^ We have suggested that evidence synthesis approaches can be used during the review stages of epidemiological studies to lend consistency and rigour to the DAG-building process. We have introduced and demonstrated ESC-DAGs as one such method. Modern and classic approaches to causal inference are combined to generate individual DAGs per reviewed study, and to subsequently synthesise them into an integrated DAG that then directs data analysis, including sensitivity analysis. ESC-DAGs is accessible to researchers with limited experience of DAGs, whereas experienced DAG users will benefit from the method’s modular design, in both using and improving upon the approach. We hope that ESC-DAGs, as a systematic, transparent, and efficient approach to building DAGs, will stimulate further debate on how researchers can use DAGs to improve population health research.

## Funding

This work was supported by the Medical Research Council [1732344 to K.D.F., MC_UU_12017/11, MC_UU_12017/13, MC_UU_12017/14, MC_UU_12017/15], the Chief Scientist Office [SCAF/15/02 to S.V.K.] and the National Institute for Health Research [11/3005/40 to S.V.K.].

**Conflict of interest:** None declared.

## Supplementary Material

dyz150_Supplementary_Data
