# Corrigendum to: Evidence synthesis for constructing directed acyclic graphs (ESC-DAGs): a novel and systematic method for building directed acyclic graphs

**DOI:** 10.1093/ije/dyz220

**Published:** 2019-10-24

**Authors:** Karl D Ferguson, Mark McCann, Srinivasa Vittal Katikireddi, Hilary Thomson, Michael J Green, Daniel J Smith, James D Lewsey

First published online: 19 July 2019, *Int J Epidemiol* 2019, doi: https://doi.org/10.1093/ije/dyz150

In the originally published version of this article, the funding section was missing. This has since been added.

