# Supplementary material for: Evidence synthesis for constructing directed acyclic graphs (ESC-DAGs): a novel and systematic method for building directed acyclic graphs
Source: Int J Epidemiol. 2019 Jul 19;49(1):322–9. doi: 10.1093/ije/dyz150 (PMC7124493; doi:10.1093/ije/dyz150)
Supplement: dyz150_Supplementary_Data [file dyz150_supplementary_data.docx]

## Appendix 1: DAG basics

### Basic DAG concepts and terminology

These examples were made in the freely available online tool ‘DAGitty’. Nodes in a DAG represent concepts or variables (a). All variables that influence at least two others in the DAG should be included. They can be measured or unmeasured. Arrows (b) can only be unidirectional with a single tail and head. They are referred to as directed edges. They indicate the direction of causation between the two nodes they connect. The basic structure of a DAG is two nodes connected by one directed edge (c). The interpretation of this structure is that the origin node in some way causes the terminus node (the effect), or that the value of the terminus node is in some way dependent on the value of the origin node(1). The DAG literature commonly employs ancestry terminology in which causes are the ancestors or parents of their descendants or children (effects)(2).

1. (b) (c)


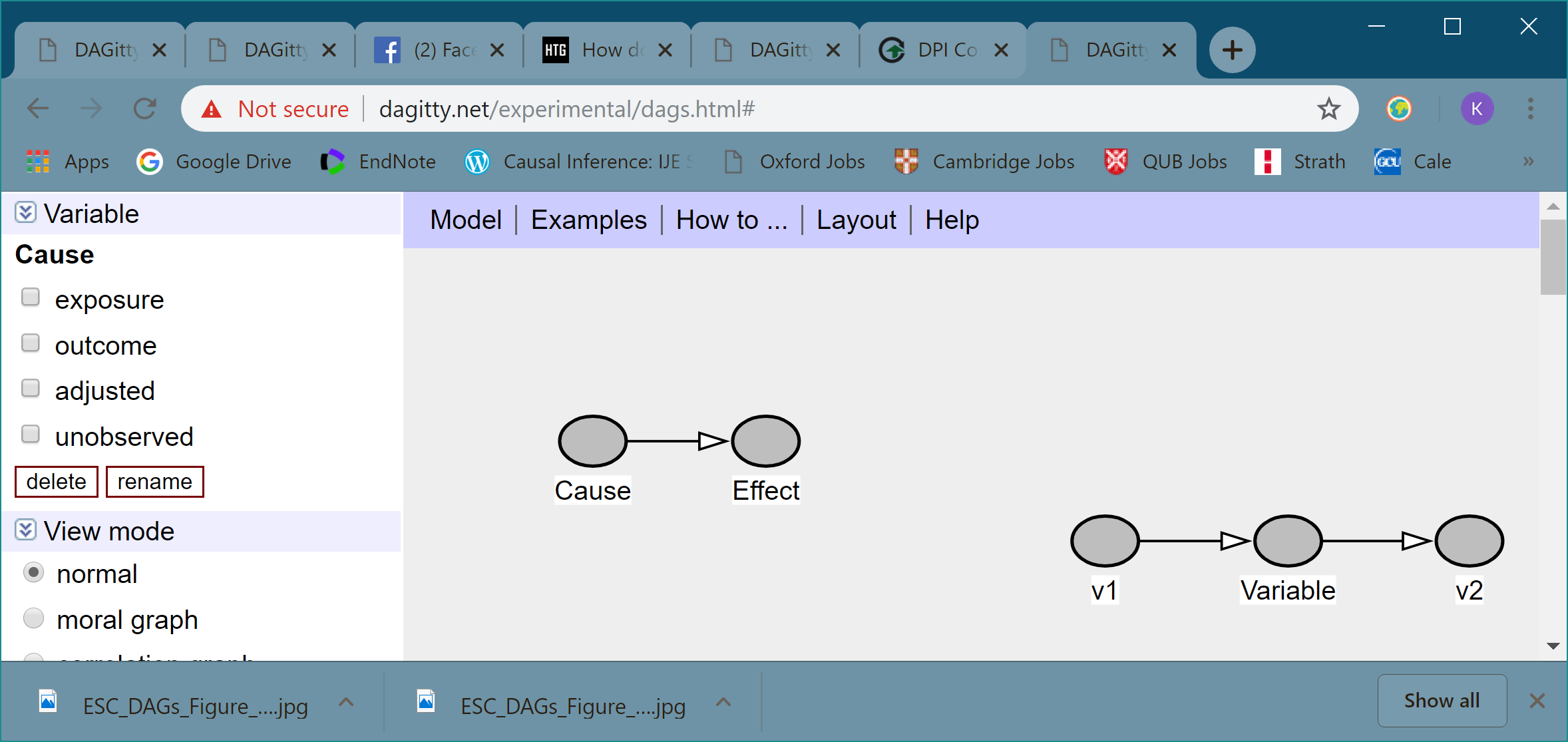

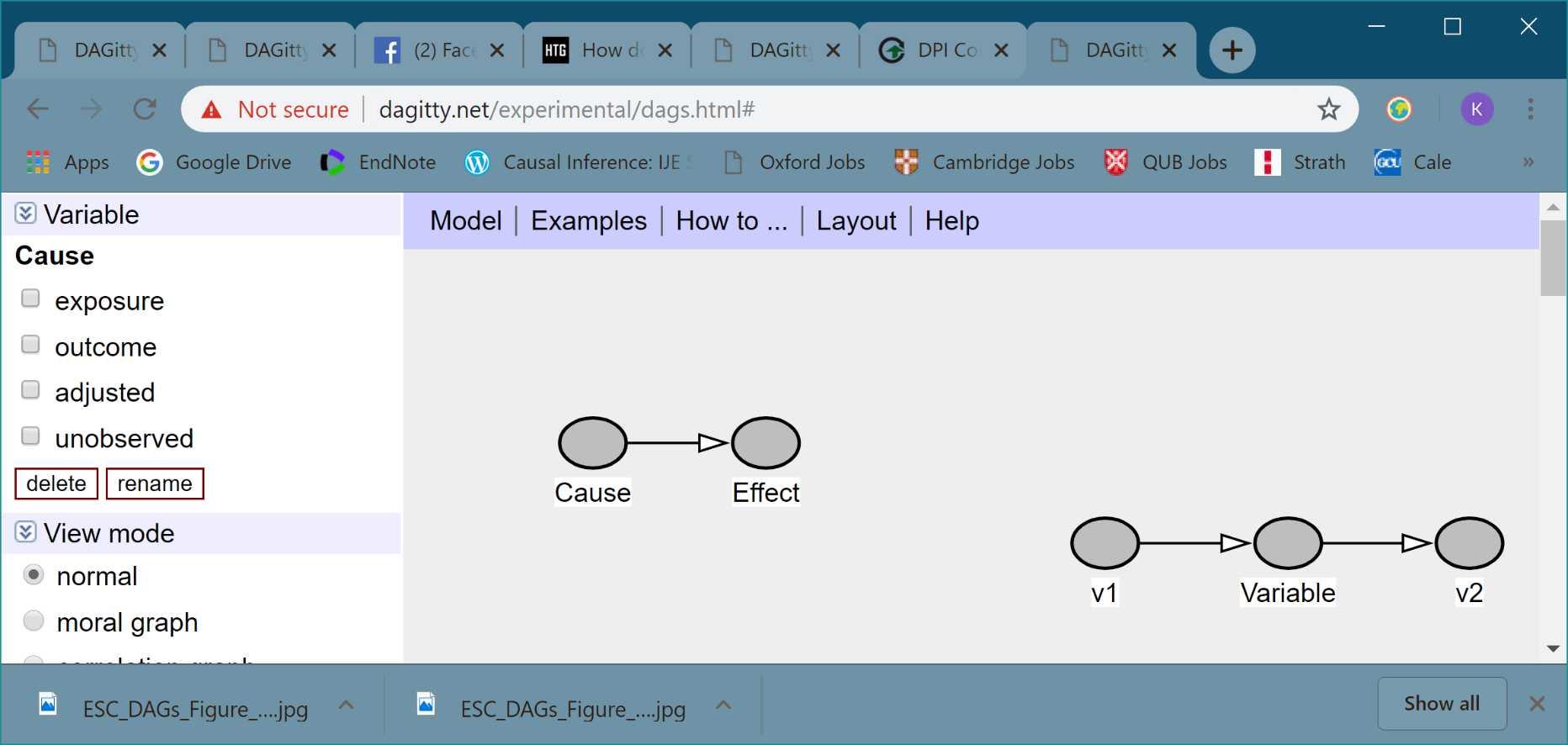

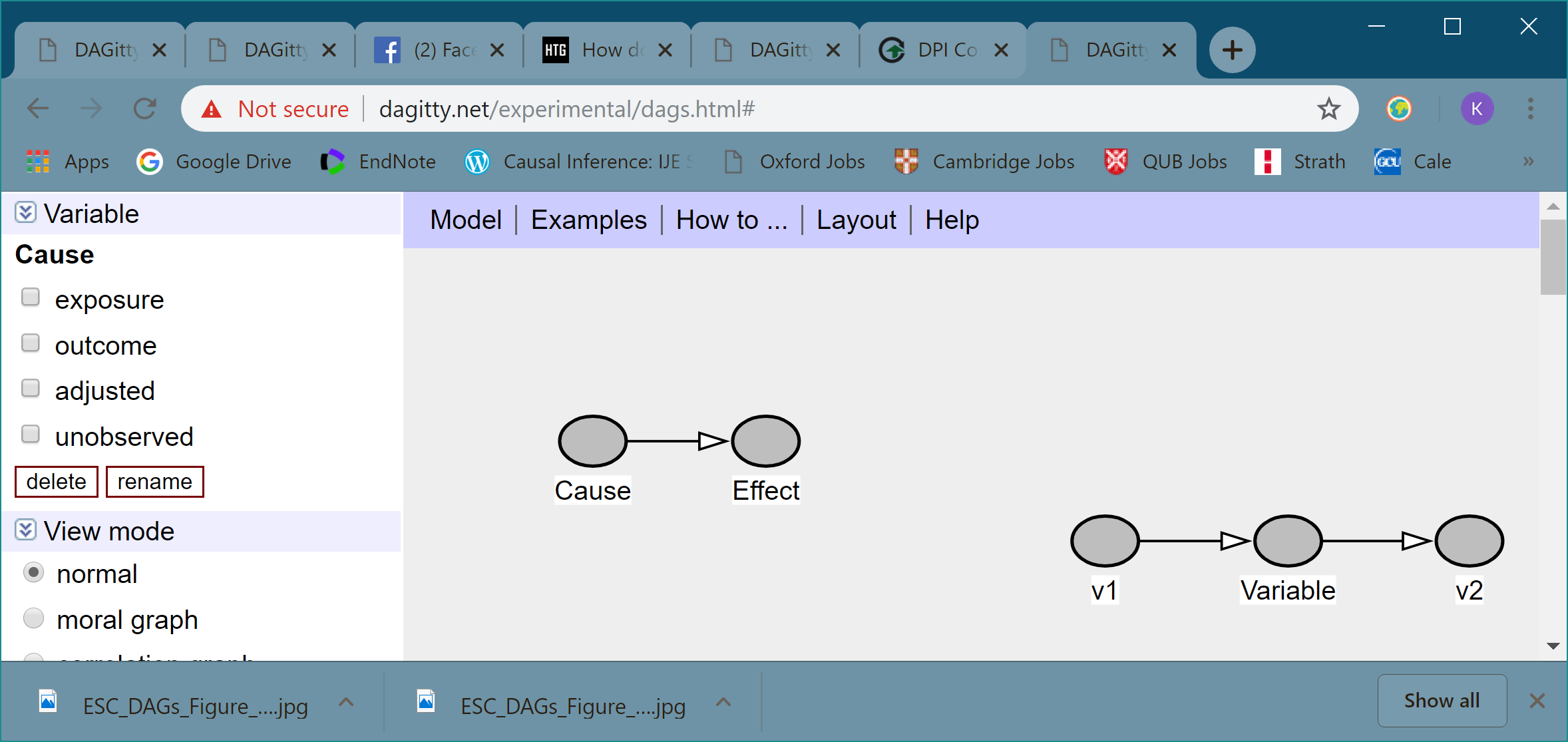


DAGs are acyclic, meaning that no combination of arrows may constitute a loop. In other words, any ‘path’ started at a particular node may not return to that node at any point. This is not to say that DAGs cannot operationalise feedback loops, but rather that they decompose feedback loops into their discrete temporal stages. In the potential outcomes literature feedback loops are often referred to as time-dependent or time-varying confounding. Further discussion is available elsewhere(3, 4).

### Causal structures and adjustment in DAGs

Mediation (d) is effectively serialised causation: at least part of the exposure’s influence on the outcome is through an indirect pathway. Adjusting for a mediator thus adjusts for part of the exposure’s effect on the outcome. A confounder (e) is a mutual cause of the exposure and outcome. As such, if researchers do not adjust for a confounder of an exposure-outcome relationship of interest, there is no way to know how much of the exposure’s effect on the outcome is due to the confounder. A collider (f) is mutually caused by 2 covariates. Adjusting for a collider induces a spurious association between its mutual causes called ‘collider bias’. Pearl and others explain collider bias in terms of the ‘back door criterion’, in that adjusting for a collider “opens a backdoor path”(1, 5). Collider bias is related to selection effects. The backdoor criterion and the related d-separation algorithm are the underlying mathematical ‘rules’ of DAGs. They are beyond the scope of this paper, but extensive discussion is available elsewhere(1, 5-7).


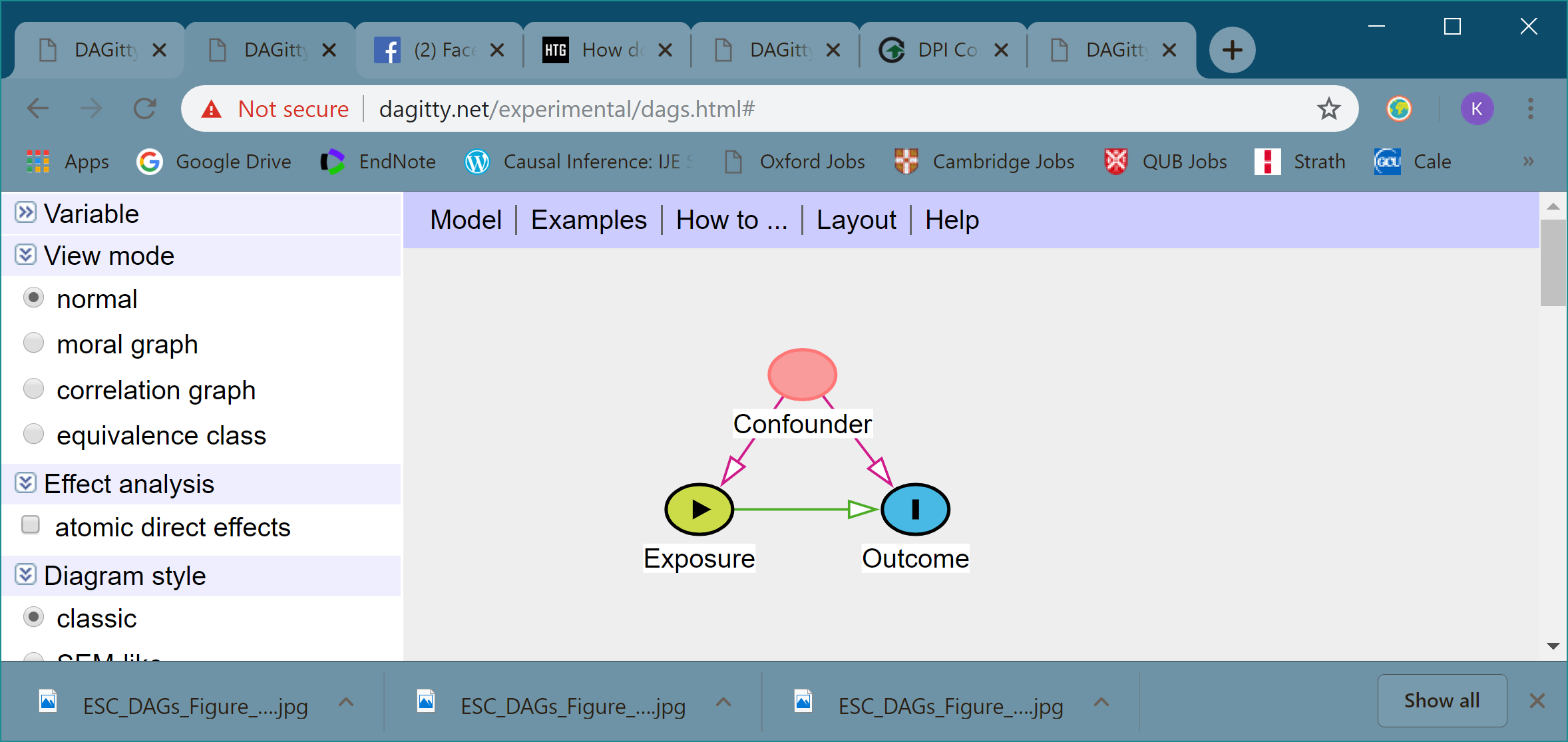

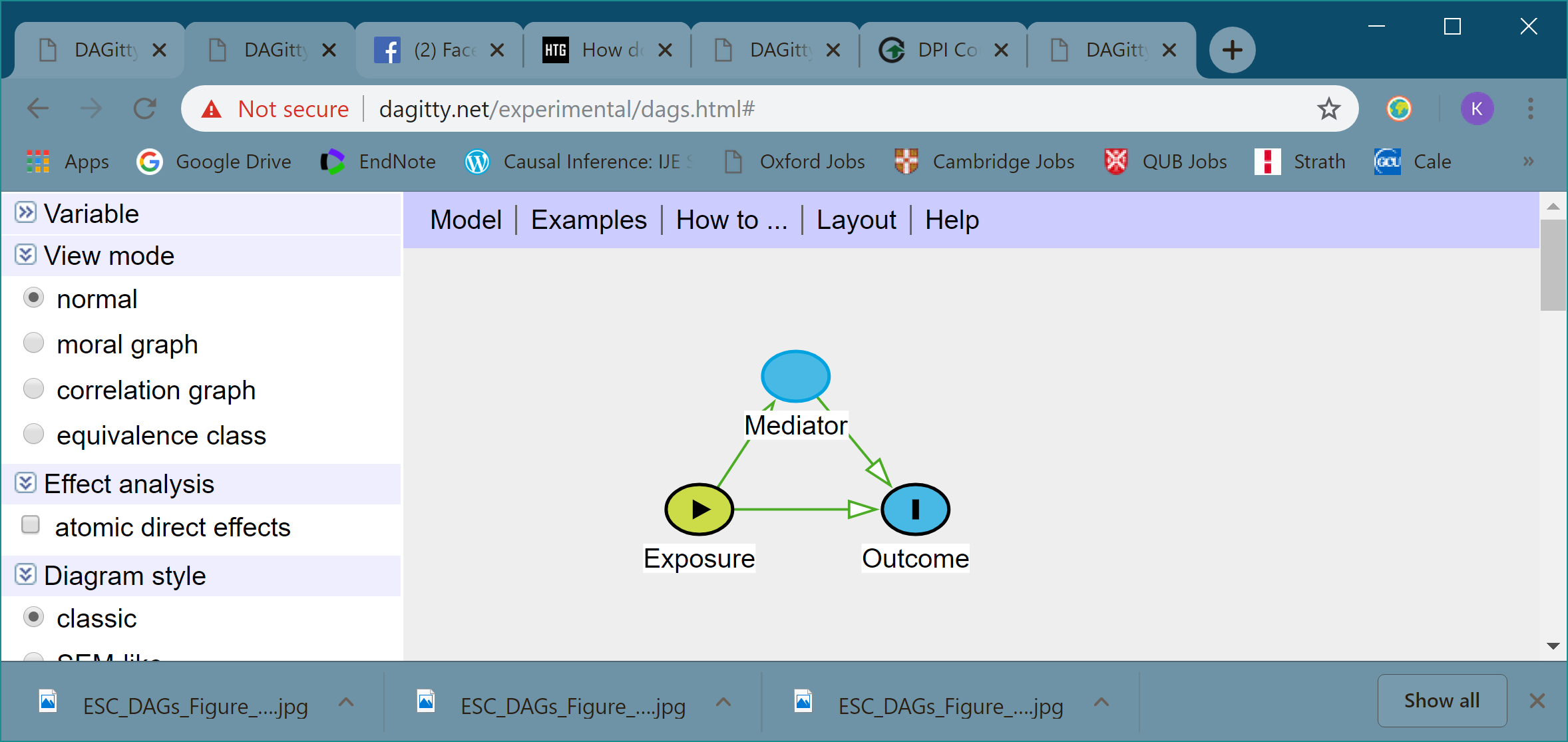
(d) (e) (f)


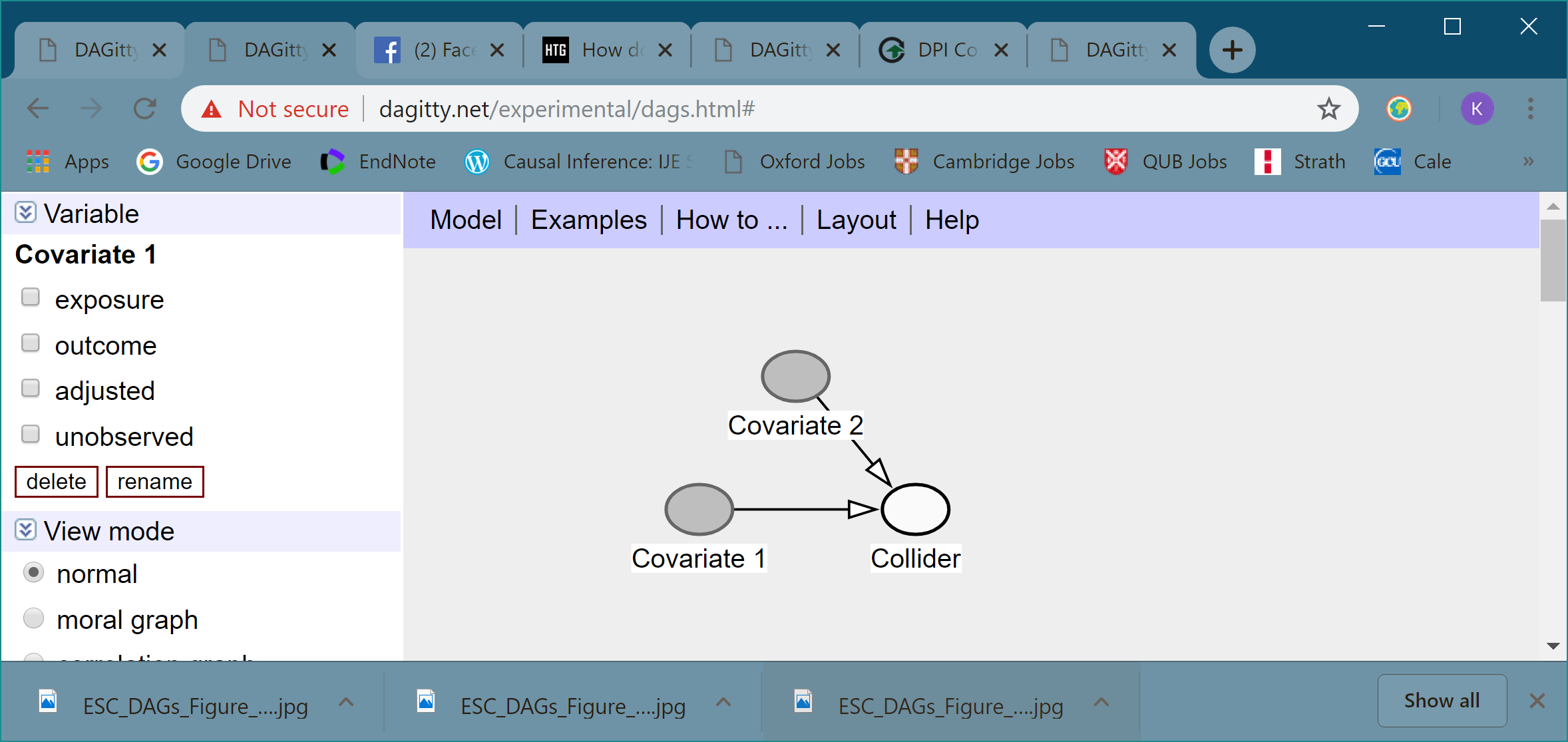


## Appendix 2: Example directed edge index

The below table depicts how the directed edge index could look. Edges for a study are compiled here once the translation stage produces the DAG for that study. In this example, the edges for the hypothetical and Seljamo et al studies have been indexed. To integrate the studies, reviewers would input these edges and nodes into a new DAG to produce an I-DAG.

**Example directed edge index**

| **Study** | **Edge originates from** | **Edge terminus at** | **Bi-directional?** |
| --- | --- | --- | --- |
| Hypothetical | Historical parental alcohol use | Adolescent alcohol use | No |
|  | Historical parental alcohol use | Adolescent substance use | No |
|  | Adolescent sex | Adolescent alcohol use | No |
|  | Adolescent sex | Adolescent substance use | No |
|  | Adolescent substance use | Adolescent alcohol use | Yes |
| Seljamo et al., 2006 | Historical parental alcohol use | Family structure | No |
|  | Historical parental alcohol use | Age of alcohol initiation | No |
|  | Historical parental alcohol use | Adolescent alcohol use | No |
|  | Family structure | Age alcohol initiation | No |
|  | Family structure | Adolescent alcohol use | No |
|  | Age of alcohol initiation | Adolescent alcohol use | No |
|  | Adolescent sex | Age alcohol initiation | No |
|  | Adolescent sex | Adolescent alcohol use | No |

## Appendix 3: Example decision log

We suggest the following items as useful for the decision log: the publication and design details for the study; the variables; the code for the IG and DAG (constructed here using DAGitty); the full assessment for edges from the IG under the causal criteria; and a record of the decisions taken for each edge. In this way the decision log is similar to a data extraction tool. The below example is an excerpt from the decision log for the Seljamo et al study, slightly simplified for demonstration purposes. It demonstrates the full causal assessment of the directed edge between family structure and adolescent alcohol use. As in the main body of the article, their study was assessed subsequent to the hypothetical study. Note that this decision log is merely a suggestion, it is not meant to be definitive. Arguably, if the goal is transparent open science, the DAGs produced for each study could be provided in lieu of their decision logs.

**Example decision log**


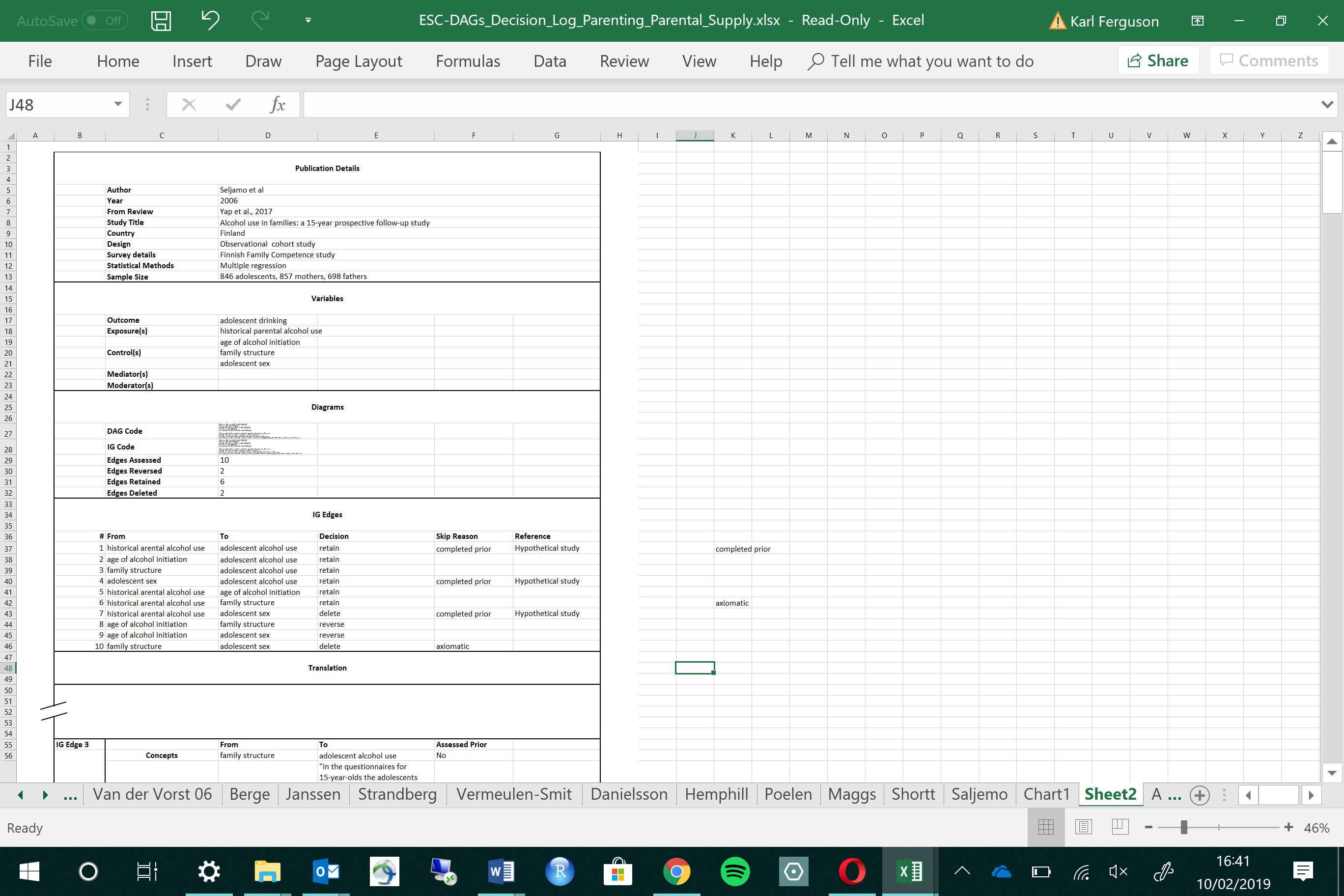


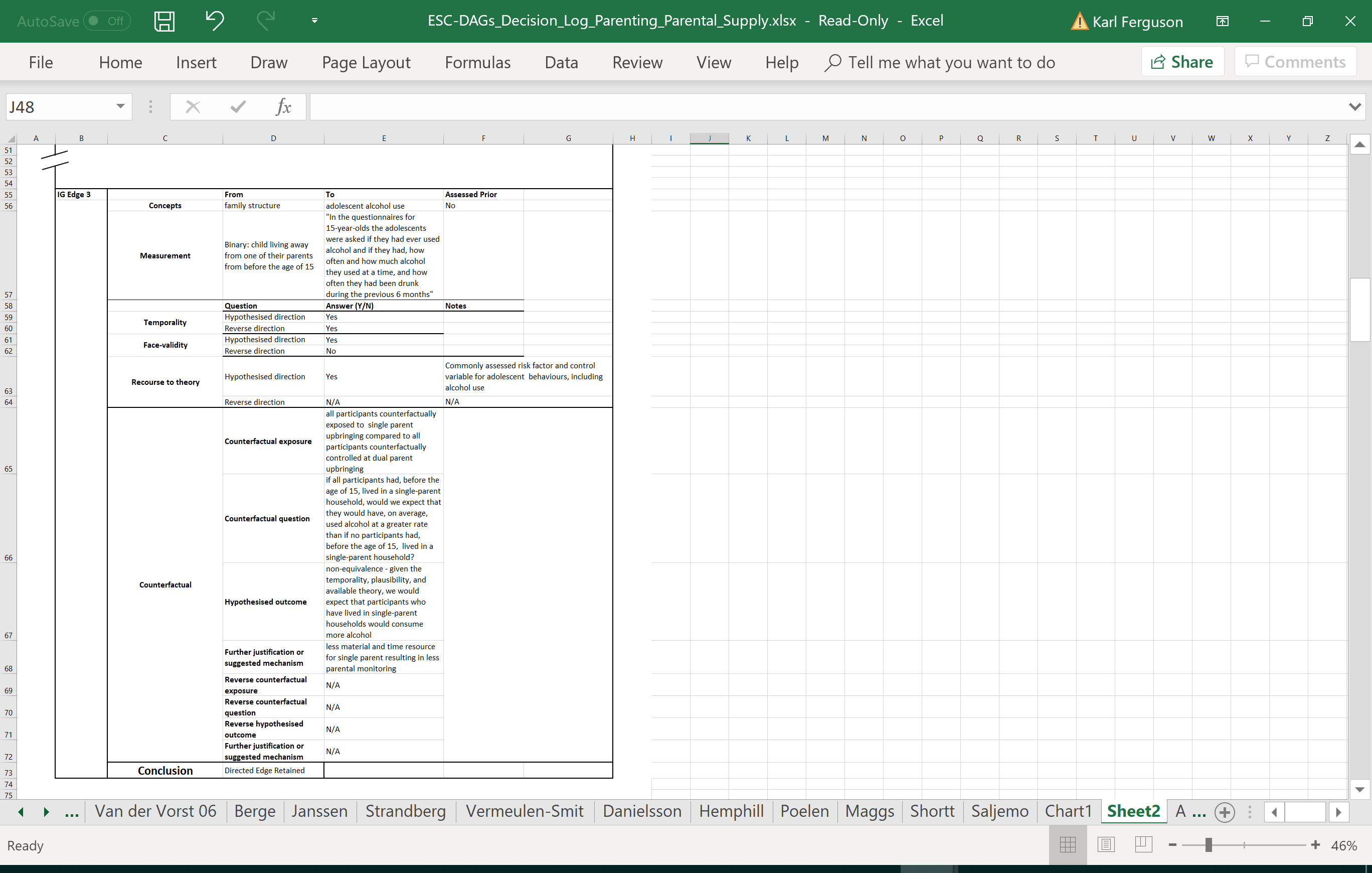


# References

1. Pearl J, Glymour M, Jewell NP. Causal Inference in Statistics: A Primer: Wiley; 2016.

2. Morgan SL, Winship C. Counterfactuals and Causal Inference: Methods and Principles for Social Research: Cambridge University Press; 2007.

3. Daniel RM, De Stavola BL, Cousens SN. gformula: Estimating causal effects in the presence of time-varying confounding or mediation using the g-computation formula. Stata Journal. 2011;11(4):479.

4. VanderWeele TJ. Explanation in causal inference: developments in mediation and interaction. International Journal of Epidemiology. 2016;45(6):1904-8.

5. Pearl J. Causality: Cambridge University Press; 2009.

6. Textor J, Hardt J, Knüppel S. DAGitty: a graphical tool for analyzing causal diagrams. Epidemiology. 2011;22(5):745.

7. Howards PP, Schisterman EF, Poole C, Kaufman JS, Weinberg CR. "Toward a clearer definition of confounding" revisited with directed acyclic graphs. Am J Epidemiol. 2012;176(6):506-11.
